# Profil épidémiologique de l'insuffisance rénale terminale à l'hôpital Militaire de Rabat, Maroc

**DOI:** 10.11604/pamj.2015.20.439.3352

**Published:** 2015-04-30

**Authors:** Mohammed Asserraji, Omar Maoujoud, Marouane Belarbi, Zouhir Oualim

**Affiliations:** 1 Dialysis Unit, First Medicosurgical Hospital, Etat Major Zone Sud, 80000, Agadir, Morocco; 2 Service de Néphrologie, Hémodialyse et Transplantation Rénale, Hôpital Militaire Rabat, Maroc

**Keywords:** Insuffisance rénale chronique terminale, hémodialyse, suivi néphrologique

## Abstract

L'Insuffisance rénale chronique terminale (IRCT) est un enjeu majeur de sante publique au Maroc à cause de ses conséquences médicales et socioéconomiques. L'objectif de ce travail est d’évaluer le profil épidémiologique de l'IRCT à l'hôpital militaire de rabat, Maroc. Il s'agit d'une enquête rétrospective pendant quatre ans (1^er^ janvier 2007 au 31 décembre 2010). Tous les cas d'IRCT (diminution permanente du débit de filtration glomérulaire (DFG) < 15 ml/min/1.73m^2^, pendant ≥ 3 mois) ont été inclus. 203 patients ont commencé la dialyse durant cette période, 130 hommes (64%) et 73 femmes (36%), l’âge moyen était de 49,92 ans (06 - 80 ans). la néphropathie d'origine indéterminée représente la première cause d'IRCT dans notre série (27,1%) suivie de la néphropathie diabétique (24,6%), des glomérulonéphrites chroniques (22,7) et de la néphropathie d'origine vasculaire (10,8%). 131 patients (64,5%) n'ont pas eu de suivi néphrologique pré dialytique. une prise en charge globale de cette pathologie est nécessaire.

## Introduction

Le profil de la morbidité et de la mortalité des maladies dans le monde entier est en train de changer, aussi bien dans les pays développés que dans les pays émergents. Les maladies non-transmissibles et non-infectieuses [1] comme le diabète et l'hypertension artérielle sont désormais la cause principale de mortalité et de morbidité. L'insuffisance rénale chronique terminale (IRCT) a été déclarée priorité majeure de sante publique dans de nombreux pays à cause d'une croissance régulière de l'incidence et de la prévalence de l'IRCT (21-22-23) et de ses conséquences médicales, sociales et économiques. Aujourd'hui, la cause principale de l'IRCT est le diabète de type 2 [2]. A la différence des pays industrialisés ou l'incidence et la prévalence de l'IRCT est connue grâce à l'existence de registre nationaux, peu de données existent dans les pays en voie de développement comme le Maroc, sur la progression des nouveaux cas d'IRCT [3]. L'objectif de ce travail est d’étudier les nouveaux cas d'IRCT à l'unité de néphrologie de l'hôpital militaire de rabat, Maroc.

## Méthodes

Il s'agit d'une enquête rétrospective, conduite au service de néphrologie hémodialyse et transplantation rénale de l'hôpital militaire de rabat, qui porte sur une période quatre ans (du 1^er^ janvier 2007 au 31 décembre 2010), au cours de ce temps, ont été recensés tous les patients atteints d'IRCT requérant la dialyse de suppléance. L'IRCT a été définie comme toute diminution permanente débit de filtration glomérulaire (DFG) au-dessous de 15ml/min/1.73m^2^ pendant plus de 3 mois. Le DFG a été calculé par la formule MDRD (modification of diet in renal desease).

Les paramètres étudies étaient: l’âge au moment du début de la dialyse, le sexe, la néphropathie causale et l'existence ou non d'un suivi néphrologique pré dialytique; le suivi en milieu néphrologique, avant la dialyse, a été étudié; au plan biologique, au moment de la mise en dialyse, ont été notes: la clairance de créatinine selon la formule MDRD, ainsi que les valeurs de l'hémoglobine, de la kaliémie, de la calcémie, de la phosphorémie, le taux de parathormone et les réserves alcalines; les comorbidités associes a l'urémie terminale: existence et ancienneté d'une hypertension artérielle (HTA), de cardiopathie ischémique, d'insuffisance cardiaque congestive. le statut sérologique (hépatite virale B et C).

Les données ont été analysées par un logiciel de statistique SPSS 10. Les variables quantitatives ont été exprimées en moyennes ± écart type est comparées par le test de « student ». Les variables qualitatives ont été exprimées en pourcentage est comparées par le test de chi2.

## Résultats

203 patients ont commencé la dialyse durant cette période (janvier 2003-décembre 2006).il s'agit de 130 hommes (64%) et 73 femmes (36%). parmi nos patients, 4 étaient des enfants. l’âge moyen de nos patients au début de la dialyse était de 49,92 ± 14,97 ans (06 - 80 ans). Il est plus élève chez les hommes (52,52 ±13,82 ans) que chez les femmes (45,27 ±15,89). L'analyse des caractéristiques démographiques et cliniques a été possible chez l'ensemble des patients.

La répartition des néphropathies causales est indiquée dans le graphique 1. la néphropathie d'origine indéterminée représente la première cause d'IRCT dans notre série (27,1%), elle est suivie de la néphropathie diabétique (24,6%), des glomérulonéphrites chroniques (22,7) et de la néphropathie d'origine vasculaire (10,8%). les autres causes d'IRCT sont représentées par ordre de décroissance comme suit: les néphropathies tubulointerstitielles (NTIC) (5,9%), la polykystose rénale (4,9%), les néphropathies obstructives (3,4%). l’âge moyen des patients atteints de néphropathie vasculaire ou diabétique est plus élève. Les données complètes sur le suivi pré dialytique sont représentées dans le Tableau 1. 131 patients (64,5%) n'ont pas eu de suivi néphrologique pré dialytique, 72 patients (38,5%) ont été suivis par un néphrologue avant leur mise en dialyse. Les données biologiques sont montrées dans le Tableau 2. On note une clairance de créatinine MDRD basse au moment de la mise en dialyse. La clairance de créatinine MDRD, le taux d'hémoglobine sont plus bas chez les patients non suivis (7,30 ± 3,30) que chez les patients suivis (8,90 ± 3,05) la différence est statistiquement significative (Test de « student »).

**Tableau 1:** Répartition des patients selon l'existence ou non d'un suivi

Existence de suivi	Nombre	Pourcentage
Non suivi	131	64,5
0-6 mois	25	12,8
6-12 mois	18	8,6
12-24 mois	5	4,5
Plus de deux ans	24	12,6

**Tableau 2:** Valeurs moyennes lors de la mise en hémodialyse

	Moyenne (± Ecart type)	Intervalle	n
Créatinine	121,15 ± 61,99	36-330	203
Clairance (mdrd)	7,94 ± 3,29	0 - 16	203
Kaliémie	5,03 ± 0,98	2,60 - 8,00	203
Calcémie	77,67 ± 14,86	29 - 115	181
Phosphoremie	52,43 ± 17,99	13 – 116	154
Bicarbonates	18,32 ± 6,43	0 – 32	100
Hémoglobine	8,31 ± 2,00	4,00 – 13,90	203
Albuminémie	30,31 ± 7,86	5 – 46	151
Parathormone	386,63 ± 254,73	23 - 978	142

**Figure 1:**
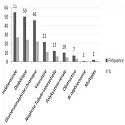
Répartition des néphropathies causales

Une hypertension artérielle a été notée chez 126 patients (62,1%), 77 patients (37,9%) sont normotendus au moment de l'inclusion. 25 patients (12,3%) ont un antécédent de cardiopathie ischémique associée à leur IRCT. Concernant les sérologies d'hépatite virales; l'antigène Hbs est positif chez 08 patients (3,9%), l'anticorps anti VHC est positif chez 06 patients (3,0%). La Figure 1 montre les principales comorbidités retrouvées.

## Discussion

La maladie rénale chronique est un enjeu majeur de santé publique, il s'agit d'un événement relativement fréquent qu'il est important de diagnostiquer tôt chez les personnes à risque et qui nécessite une prise en charge adaptée selon le stade de gravite et l’âge des patients [4]. Selon l'organisation mondiale de la santé (OMS), l'IRCT constitue actuellement la 12^eme^ cause de mortalité et la 17^eme^ cause de morbidité dans le monde. Cette affection est silencieuse, lentement évolutive et responsable d'une augmentation du risque cardio-vasculaire. C'est aussi une pathologie fréquente et en constante progression [5, 6]. Peu de données existe au Maroc concernant l’épidémiologie de l'IRCT, son incidence, calquée sur les pays maghrébins, est estimée entre 100 et 150 nouveaux cas (nc)/10^6^ d'habitants/an (soit ≈ 3000-4500 nouveaux cas/an), sa prévalence a 167 cas/10^6^ d'habitant (le nombre de patients actuellement hémodialyses ≈ 5000). Il faut souligner que 80% des IRCT ne sont pas prises en charge en dialyse [7].

En dépit du son caractère monocentrique et de la faiblesse de l’échantillon, notre étude est une approche épidémiologique de cette pathologie lourde est couteuse. Une étude comparable réalisée en 2003 dans la ville de Casablanca s'est intéressée à 279 patients atteint d'IRCT et pris en charge per un organisme mutualiste montre aussi la place du diabète comme principal pourvoyeur de l'IRCT [8]. Le registre national de recueil de données sur l'IRCT, MAGREDIAL (Maroc greffe dialyse) qui est actuellement fonctionnel, représente une politique sanitaire adapte à ce problème de santé publique.

Plusieurs études ont suggère qu'une durée suffisamment prolongée du suivi néphrologique s'accompagne d'une meilleure préservation de l’état cardiaque et vasculaire des patients. [9, 10]. Malheureusement, sur le plan pratique, la mise en dialyse s'opère dans des conditions de fortes comorbidités. Nous constatons dans notre étude les mêmes faits, parfois dans des proportions plus importantes. En effet, au moment de leur mise en hémodialyse, 62% des patients sont hypertendus, 12,5% ont une cardiopathie ischémique, 2% ont une artérite des membres inférieurs, 1,2% ont une insuffisance cardiaque compromettant la mise en hémodialyse et enfin 8,4% ont une hépatopathie.

## Conclusion

Au Maroc, la maladie rénale chronique (MRC) et son corollaire l'IRCT représentent un vrai problème de santé publique nécessitant une prise charge globale faisant intervenir l'ensemble des acteurs de la sante publique.
